# Mindfulness and Understanding of Self-Care for Leaders of Extension: Promoting Well-Being for Health Educators and Their Clients

**DOI:** 10.3389/fpubh.2022.862366

**Published:** 2022-05-13

**Authors:** Anna Dysart, Samantha M. Harden

**Affiliations:** Department of Human Nutrition, Foods, and Exercise, Virginia Tech, Blacksburg, VA, United States

**Keywords:** dissemination, implementation, RE-AIM evaluation framework, mindfulness, online intervention, employee wellness

## Abstract

**Background:**

Mindfulness and self-care, practiced through a variety of methods like meditation and exercise, can improve overall sense of holistic well-being (i.e., flourishing). Increasing mindfulness and self-care may lead to increased flourishing and job satisfaction among the nation-wide Cooperative Extension system delivery personnel (agents) through a theory-based online program and an extended experiential program.

**Methods:**

Cooperative Extension agents from two states were invited to participate in MUSCLE *via* statewide listservs. Participants were invited to attend sessions and complete competency checks and between-session assignments each week. The study was conducted using Zoom. Pre- and post- program surveys included validated scales for flourishing and physical activity status. Due to high demand for mindfulness programing during the onset of the COVID-19 pandemic, experiential “Mindful Meet-up” 30-minute sessions were held on Zoom. Dissemination and implementation of the two differing interventions (i.e., MUSCLE and Mindful Meet-ups) were examined.

**Results:**

MUSCLE (more intensive program with assignments and competency checks) had lower reach, and did not show statistically increased flourishing or physical activity. Mindful Meet-ups had higher attendance and proportional reach during the beginning of the pandemic, but no practical measure of flourishing or physical activity behaviors. Unsolicited qualitative feedback was encouraging because the interventions were well-received and participants felt as though they were more mindful.

**Conclusions:**

While agents anecdotally reported personal improvements, capturing data on outcomes was challenging. Complementing outcome data with implementation and dissemination outcomes allowed for a richer picture to inform intervention decision-making (i.e., offering the same or new programming depending on participant needs).

## Introduction

Physical, mental, and emotional health are all components of individual well-being, also called flourishing, and health status (1). Flourishing is considered a holistic approach to well-being as it is considers six domains: Happiness and Life Satisfaction, Mental and Physical Health, Meaning and Purpose, Character and Virtue, Close Social Relationships, and Financial and Material Stability (1, 2). There has been increasing interest in flourishing and other holistic approaches to employee wellness (3–5), and in fact has been deemed a new wave of public health (6). Interest in the promotion of human flourishing within worksites is unsurprising since work and job life are areas where individuals spend much of their time (2). There are no recognized formal guidelines for increasing flourishing, although work stressors are increasingly being recognized as public health concerns, especially for those in service positions (from healthcare providers to social workers) (7, 8). Therefore, a worksite-based wellness intervention focused on improved flourishing may reach a large number of people and have downstream impacts on the workforce ability to provide public health interventions.

One workplace of interest is the Cooperative Extension system (herein: Extension). Extension is a nation-wide, federally funded system where educators work to engage with the community and provide education and programming to meet community needs (9–11). Within Extension, educators (most often called agents) have a unique set of skills and competencies (12, 13) to disseminate public health programs and interventions (9, 10). Similar to other worksite wellness investigations, (2, 14, 15) is known that Extension personnel who have higher job satisfaction are more motivated and high performing (2, 14). Incorporating stress management ideals and other aspects that promote overall flourishing (1) within wellness initiatives can also result in higher job satisfaction, (2, 15) lower levels of job stress, (15) and lower intention to leave (14). One well-studied intervention that can help promote lower levels of stress is yoga and its mindfulness principles (16–20).

Yoga is thought to reduce stress through utilizing breathing and movement patterns (19, 20). Yoga can be defined as an ancient mind-body practice in which the flow of movement is matched to the breath, though there are a wide range of yoga styles and some yoga practices may only incorporate breath work (21, 22). The Physical Activity Guidelines for Americans (PAG) (23) recommendations include yoga as a multicomponent physical activity (those that include muscle strengthening, aerobic, and balance activities) and as relative moderate-intensity activity for some groups of individuals (23). Yoga has been studied within multiple settings, including a university workplace (19), healthcare workers (3, 24), and graduate students (5) as a method of stress reduction, and was found to reduce stress and anxiety. Example of “yoga” interventions include yoga sessions focused on mindful awareness of posture and breath (3), yoga sessions focused more on the physical practice of yoga (16), or yoga sessions with a combination of didactic learning and experiential breath and movement (5). The success of yoga within these varying work fields suggests it may also be successful within an Extension system wellness program to promote flourishing.

In yoga, “mindfulness” can be an umbrella term to align with pratyahara (withdrawal of the senses), dharana (concentration), dhyana (meditation) and samadhi (absorption). In public health interventions, mindfulness has been defined as being fully aware and present in the moment. Examples of mindfulness interventions include breath and postural awareness practices (3), mindfulness-based stress reduction, and meditation (5), where participants are asked to be consciously aware of their body and breathing and may include focus on a positive word or phrase. Mindfulness interventions, both based within yoga interventions and not, has also been found to be an appropriate and effective method for stress reduction (5, 25, 26) and burnout reduction (3). Participants in an 8-week yoga-based mindfulness intervention were found to have decreased emotional exhaustion and increased mindfulness (3). Interventions with mindfulness-based focused were also found to be beneficial in decreasing anxiety and stress (3, 5), and increasing self-compassion and mindfulness (5). Yoga can also help bridge self-care interventions, with reduced burnout and increased self-care (3).

Self-care has been defined as behaviors and processes that maintain overall health through health promoting practices and managing signs and symptoms (27), and it may encompass strategies such as nurturing interpersonal connections, performing emotional hygiene (24), and physical movement like yoga (3). For example, increased self-awareness through attention to typically unthought of bodily functions like breathing which may increase ability to handle stress (3). Including self-care within workplace wellness interventions may help to diminish stress (5), with less stressed employees having higher performance and better job satisfaction (2, 15).

Taken together, it is known that physical activity in the form of yoga, mindfulness, and self-care can all play a role in reducing stress (3, 5, 19, 20, 24). Yet, much of what is known about these interventions comes from explanatory, or more controlled, clinical trials. A pragmatic approach to research takes into account the stakeholder and community input and can help accelerate putting interventions into practice (28). RE-AIM is a frequently used planning and evaluation tool that has 5 dimensions—*r*each, *e*ffectiveness, *a*doption, *i*mplementation, and *m*aintenance (29). Use of RE-AIM in pragmatic planning and evaluation (28, 30, 31) provides a full picture of the interventions to inform and improve public health.

Therefore, it was the purpose of this work to explore an iterative adaptation of a flourishing intervention for Extension employees. Specifically, it was hypothesized that a workplace wellness intervention that incorporates yoga might be able to encourage increased physical activity, mindfulness, and self-care. To explore this hypothesis, mindfulness and self-care interventions were developed and deployed using the RE-AIM framework (32).

## Methods

### Overview

The Physical Activity Research and Community Implementation (PARCI) Laboratory at Virginia Tech serves the Commonwealth of Virginia in two ways: (1) identifying Extension agent needs (and subsequently providing training, evaluation tools, and ongoing support) and (2) matching evidence-based programming with county resident needs. PARCI lab members meet with agents representing each of the four districts of the state and, as a team, decide programming needs for the following year (33–36). In 2020 it was determined that agents were in need of strategies to improve their own flourishing. Their own stress or lack of self-efficacy was hindering them from selection, adoption, and delivery of yoga-based health promotion programming. The series of programming described here was a result of these efforts. Members of the PARCI lab needed to ensure that these efforts were having a positive impact. The iterative Assess, Plan, Do, Evaluate, Report (APDER) cycle of RE-AIM, outlined by Harden et al. (29) was applied to capture the overall impact on the promotion of flourishing among Extension professionals. This cycle allows the team to iteratively capture the RE-AIM outcomes, make a plan for improvement where necessary, and inform intervention decision-making.

RE-AIM dimensions were operationalized as the following: The reach of an intervention or program is how well it connects with the intended audience (32). This allows investigators to know what they might need to do differently in order to draw more people to the program. Effectiveness is the impact the intervention had on the target health behavior. The reach and effectiveness in a program are both very important in getting a system or target staff to agree to adopt the program. The program implementation, in terms of cost, and adherence to the original intervention, is also an important factor in both the adoption and maintenance, or ongoing intervention effects, of the program (32). Each program was subsequently charted on a scale of 1–5 (1 being “none” and 5 being the “largest reach”, “more effective”, etc.) for each dimension of RE-AIM to develop program comparison of intervention component strengths and weaknesses.

**Figure 1:**
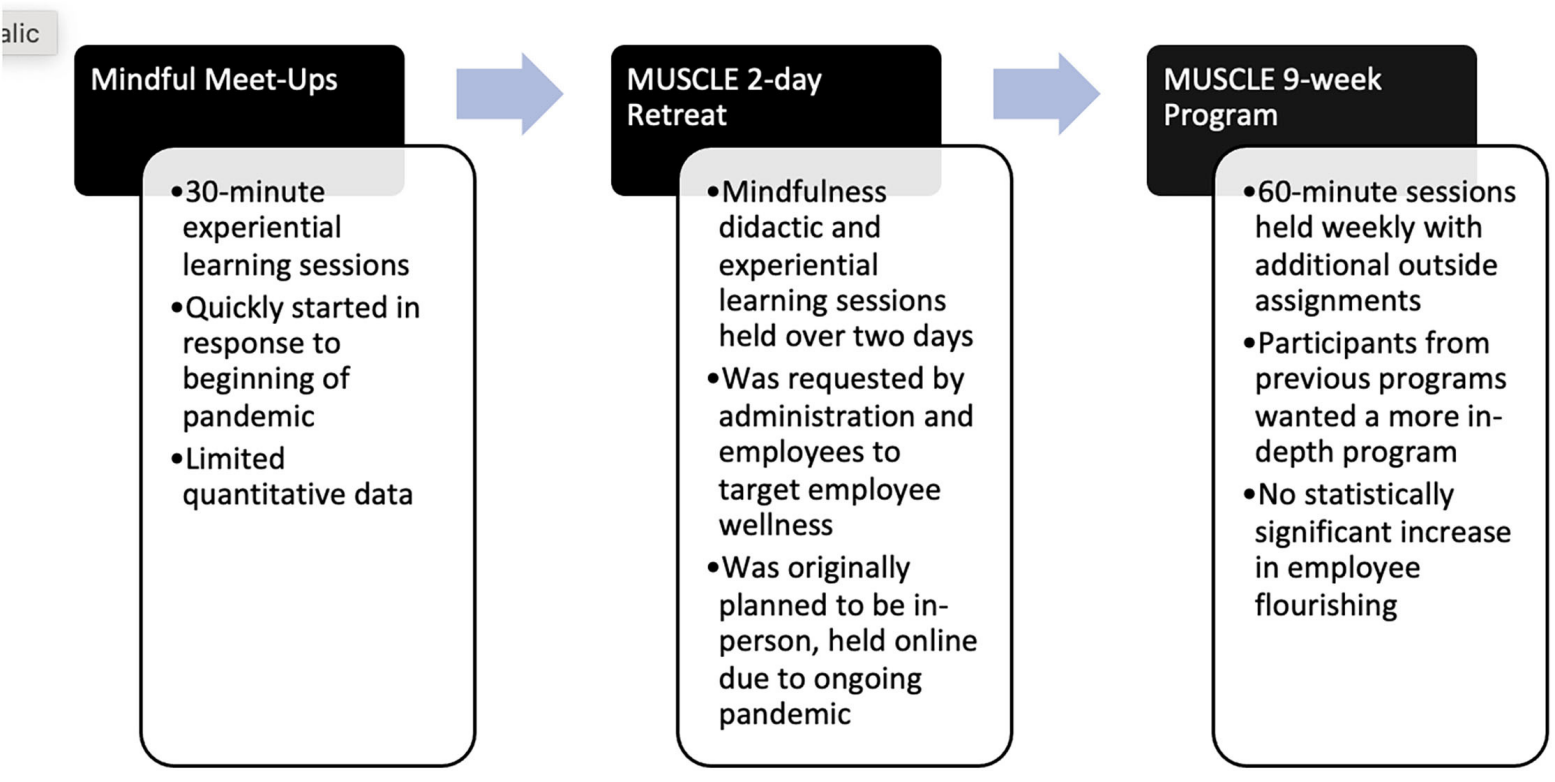
Chronological development of mindfulness programs.

Phase I. In the spring of 2020, with the work from home orders due to the COVID-19 Pandemic, 30 minute “Mindful Meet-up” sessions were scheduled for Monday mornings on the video conferencing platform, Zoom (see Figure 1). These sessions consisted of both lecture and experiential learning about a variety of mindfulness and stress-relieving tips for working from home as well, agents were provided with an informational and educational infographic that the agents could use after the sessions. Session themes included breathwork, different types of meditation, and a variety of self-care ideas such as getting outside or getting more physically active. All programs were led by individuals who were registered yoga teachers and trained in mindfulness practices, including meditation and breathing. After a semester of programming, ongoing efforts were requested by agents and Extension administration.

Phase II. In the fall of 2020, with the continued pandemic, a 2-day mindfulness and self-care retreat titled “Mindfulness and Understanding of Self-Care for Leaders of Extension (MUSCLE)”, was planned to be online (in place of the originally planned in-person retreat). Sessions included experiential physical activity opportunities such as yoga, tai chi, strength training, and interval training, as well as mindfulness opportunities such as sound bath healing, journaling, and self-compassion sessions. Other sessions were didactic and explored less typically acknowledged self-care and physical activity options like gardening tips, hiking, cooking sessions, and tips for working from home.

Phase III. To meet the requests for more education on the topic and to follow-up from other programs on physical activity (described more below), a 9-week version of MUSCLE was conducted in the fall/winter of 2020. These sessions included covering the Physical Activity Guidelines for Americans, how yoga can fit into the guidelines, yoga myth busting, what mindfulness and self-care can encompass, mindfulness and breathing techniques, self-care techniques, and scope of practice and cueing techniques for personnel who might incorporate these practices into their own work. Since there was more time prior to the launch of this program, research questions were formulated and validated surveys were used during program registration (described below).

The Phase III MUSCLE program was the most intensive of the three phases. The program was conducted at 12pm EST for one hour *via* Zoom for nine consecutive Mondays. Each synchronous session included both didactic and experiential learning. Between sessions attendees were asked to complete asynchronous activities for mastery experiences related to techniques of mindfulness and self-care. These mastery experiences were included as practice as it has been shown that it promotes improved outcomes (37, 38). For audit and feedback, asynchronous activities were discussed further in breakout rooms during the next Zoom session, or put on the competency checks completed by participants.

Between synchronous sessions, support emails were sent to the entire participant pool that included a brief summary of the information covered during the synchronous session, the recording of the instructor view of the session (not the breakout rooms), as well as the activity to complete for the asynchronous portion of the week.

To facilitate behavior change, group dynamics strategies (39) and individual behavior change strategies were utilized. These included methods such as breakout rooms on Zoom within the synchronous sessions to enhance collective efficacy, competition, and group communication/interaction. Groups within the breakout rooms were sized to promote interaction and cohesion, with approximately 5 participants per group. Groups came up with team names to promote group distinctiveness. Individual behavior change strategies included having social support from other participants, encouraging the use of action & coping plans, and having participants utilize self-monitoring tools as part of their asynchronous learning (40).

### Recruitment and Participants

Phase I. Virginia Cooperative Extension employees were invited to attend Mindful Meet-ups *via* an Extension employee email listserv a few weeks after employees received work-from-home orders in response to COVID-19. Subsequent invites were sent out *via* email during each semester that the program was held. Most attendees were from the Family Nutrition Program or the Food and Consumer Science divisions of Extension. Any employee could attend from registering on Zoom, no data were collected (with the exception of the number of attendees) and no human subjects research was sought or approved.

Phase II. Virginia Cooperative Extension employees were invited to attend the MUSCLE 2-day retreat *via* an Extension employee email listserv. Participants were able to attend some or all of the sessions based on their own interests and availability. Registration consisted of completing an interest survey for which sessions the employees wanted to attend, demographic characteristics, the L-CAT (41), the Vanderweele Flourishing scale (1), and YSES (42). This was deemed IRB human subjects exempt.

Phase III. The MUSCLE 9-week program was a follow-up to a previously completed physical activity micro-credentialing program that was open to both Virginia Cooperative Extension and Arkansas Cooperative Extension. All employees who attended the physical activity micro-credentialing program were eligible to attend MUSCLE sessions. They were verbally invited to participate in MUSCLE during the last week of the Physical Activity in Cooperative Extension program as well as *via* email (Appendix A). The email was sent out twice, once 30 days before the study sessions began, and once 2weeks before the study sessions began. There were 130 PACE registrants (65 from each state) eligible to participate in MUSCLE. Registration consisted of completing an interest survey for which sessions the employees wanted to attend, demographic characteristics, the L-CAT (41), the Vanderweele Flourishing scale (1), YSES (42), and consent. This was approved by the University IRB as human subjects research.

### Measures

The iterative APDER cycle was applied throughout the phases of this project. The needs of the Extension employees had been assessed and wellness programs were planned and delivered. The focus of these initiatives was service (in response to agent needs). However, with ongoing success within the reach dimension (Phase I), interest in more systematic evaluation was applied for Phase II (MUSCLE 2-day retreat) and Phase III (MUSCLE 9-week program). This included qualitative and quantitative data collection as outlined below. In addition, each phase was compared in terms of relative reach, effectiveness, adoption, implementation, and maintenance on a 5-point Likert scale (32) (see Figure 3).

### Reach

#### Sociodemographic Variables

Participants were asked to disclose their age and how many years they had worked with or been affiliated with Cooperative Extension during Phase II and III. The hypothesis was the years working with Cooperative Extensions would correlate positively with flourishing and confidence in delivering physical activity.

### Effectiveness

MUSCLE participants were given pre- and post-program surveys to complete, whereas the Mindful Meet-up participants were given surveys at the beginning of the third iteration/semester of the program and post-program surveys at the end of the third semester.

#### Flourishing

Flourishing is when all sectors of a person's life are good and there is a sense of well-being (1). To measure this construct the VanderWeele Secure Flourishing Index (SFI) (Appendix B) that breaks flourishing into six domains was used at pre, post, and follow-up (2). Each domain has two questions on a 0–10 scale. The domains were: Happiness and Life Satisfaction, Mental and Physical Health, Meaning and Purpose, Character and Virtue, Close Social Relationships, and Financial and Material Stability (1). The last domain (Financial and Material Stability) was included not as a goal for current flourishing, but as means to sustain flourishing (2). Each domain has two questions on a 0–10 scale. Scales included anchors of “Not Satisfied at All” to “Completely Satisfied”, “Not True of Me” to “Completely True of Me”, “Poor” to “Excellent”, “Not at all Worthwhile” to “Completely Worthwhile”, “Strongly Disagree” to “Strongly Agree”, “Not True of Me” to “Completely True of Me”, and “Worry All of the Time” to “Do Not Ever Worry”. Higher scores on the index correlate with increased flourishing. The SFI was validated for use in a workplace setting in 2019 by Weziak- Bialowolska, et. al with the correlation that workers who experience increased flourishing also experience increased job satisfaction and work engagement (2).

#### Self-Efficacy for Yoga

The yoga self-efficacy scale (YSES) (Appendix C) is a validated tool used to assess the confidence a participant has in their yoga practice and their sense of competency in their yoga practice (42). The YSES is a 12-item tool with each item rated on a nine point Likert scale with anchors of strongly disagree to strongly agree. The 12 items are broken down into three domains—Body, Breath, and Mind. Higher YSES scores correlated with increased health competence and health-related quality of life during the validation study. While the YSES had the highest Cronbach's alpha for practitioners of the target yoga style, the YSES was shown to still be valid for practitioners of five other common yoga styles (42). It was hypothesized that yoga self-efficacy, particularly the breath and mindfulness components, would be increased following the mindfulness interventions, which included yoga components.

#### Physical Activity

The Stanford Leisure-Time Activity Categorical Item (L-CAT) (Appendix D) is a validated, one item tool with six differentiating responses to assess non-work physical activity (41). It has been validated with both overweight/obese men and women (41, 43), utilizing both pedometers (44) and Sensewear^TM^ Armbands (43) as comparative assessments of physical activity. It has also been compared to the Godin Leisure Time Exercise Questionnaire with populations of low health literacy and shown to have similar results (44). The L-CAT version 2.2 was used in this study, which was shown to be accurate when compared with other measures of physical activity, (43) and is the suggested version to use by the creators of the L-CAT (43). Statements with higher numbers corelate to increased activity (41).

**Figure 2:**
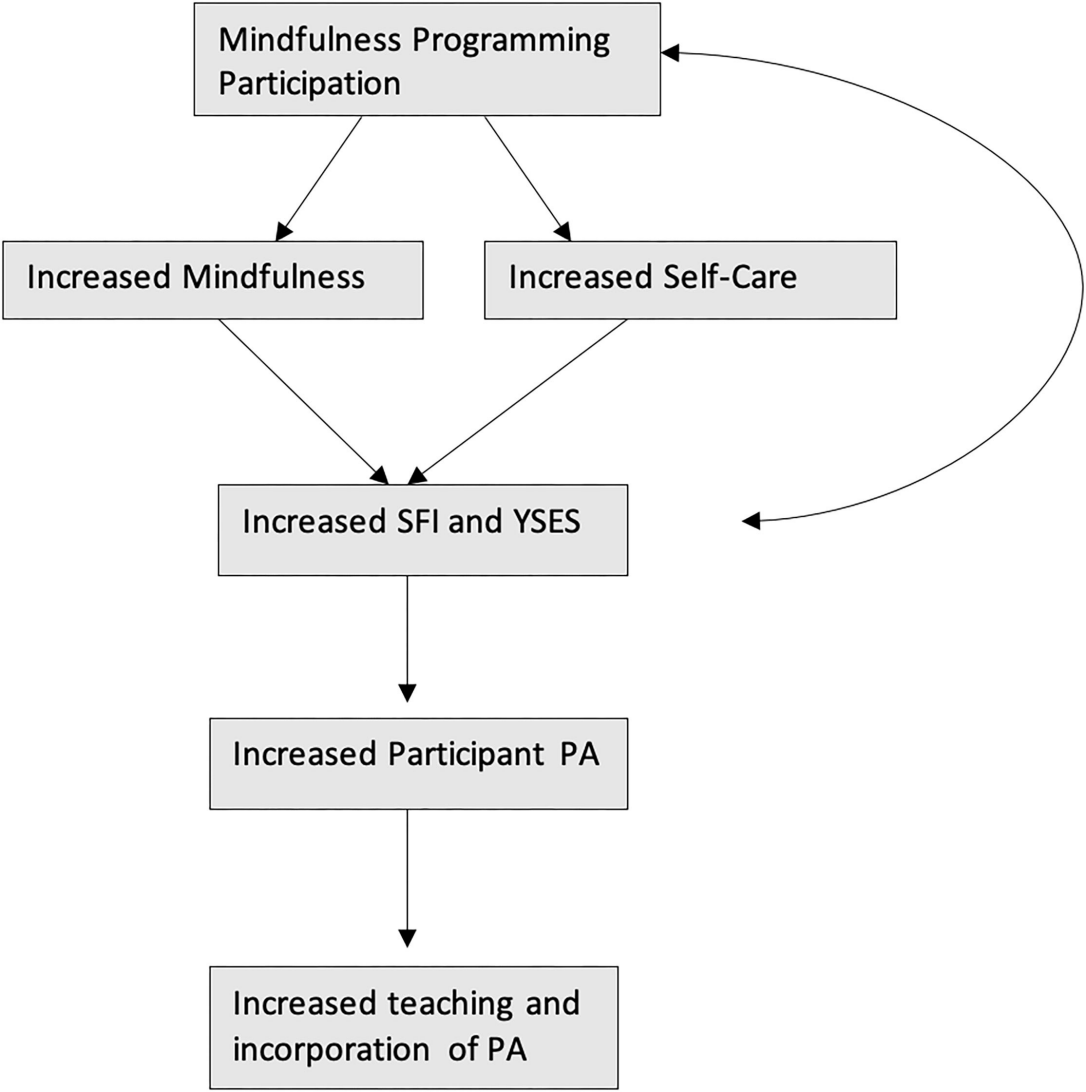
Proposed pathway for increasing mindfulness and self-care.

Separate questions were asked of the participants to determine the proportion of time during work hours the participants spent with Cooperative Extension participants conducting physical activity. The working hypothesis, based on the social cognitive theory for health promotion, (4) was that attendance of MUSCLE and Mindful Meet-up would start a chain of increased learning and knowledge that would end with increased teaching of physical activity to Cooperative Extension participants and increased incorporation of PA within Cooperative Extension workplaces (Figure 2).

#### Competency Checks

At the end of each week of the MUSCLE program, participants were asked to complete competency checks *via* Qualtrics software. The competency checks were de-identified, with participant IDs being their team name and a randomly chosen number that they were asked to decide upon in their first breakout room during the first Zoom session. Competency checks included information checks on the information covered during the synchronous sessions, as well as the asynchronous activities the participants were to complete throughout the week. By completing the competency checks, participants had another point of contact that encouraged them to complete their self- monitoring tools, action and coping plans, etc. Competency checks were evaluated based on completion, and not based on percent correct answers due to the subjective nature of some questions, i.e., “What is your favorite method of self-care?” This follows the projected model that increased MUSCLE participation will increase SFI and YSES, and results of increased PA will be seen on the follow-up L-CAT surveys.

### Adoption

Adoption was assessed as the number of teams or leaders outside of the PARCI Lab that chose to lead sessions or expand the programs during Phase I, II, or III. Additionally, strategies to increase adoption by other systems within the university or Extension systems were employed. These strategies included meeting with other teams and leaders who aimed to start employee wellness programs, providing support, and having those leaders and teams lead sessions in the Phase I and II programs.

### Implementation

Each program was implemented through online video conferencing software. Calendar invites for each program were sent to registered participants to encourage attendance post-registration. Hours of time team members worked to implement each program was specifically tracked for MUSCLE and more generally estimated for Mindful Meet-ups.

### Maintenance

Follow-up surveys were sent to each participant at the conclusion of the MUSCLE program and Mindful Meet-ups semester series, as well as 6 months after the conclusion of the MUSCLE program. Follow-up surveys included the same validated survey items from the pre-survey, including the SFI, the YSES, and the L-CAT.

### Analysis

**Figure 3:**
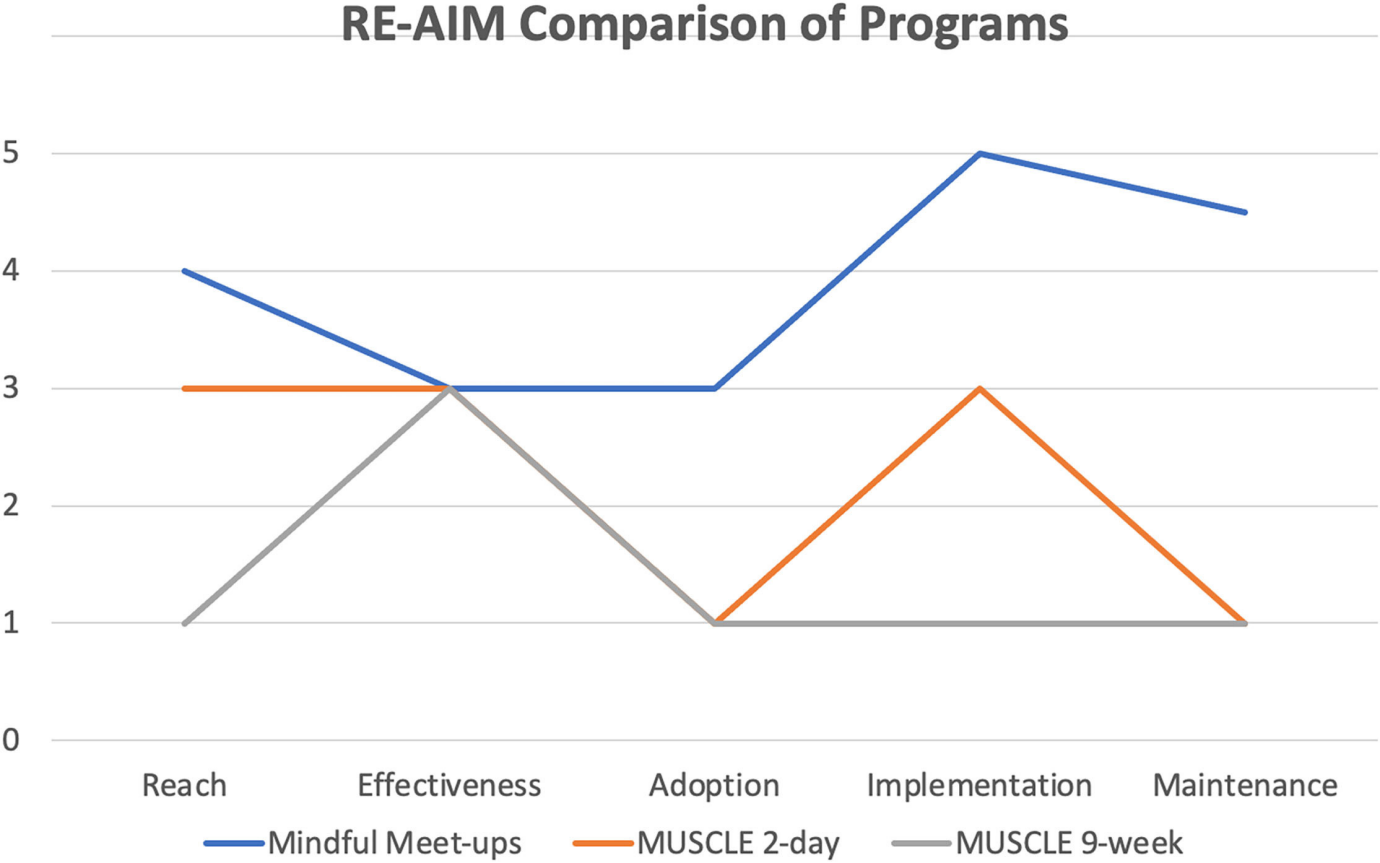
Comparison of 3 mindfulness programs across the 5 dimensions of RE-AIM.

Descriptive and inferential statistics for all data were calculated using SPSS software (IBM, version 26). Paired *t*-tests were used to evaluate physical activity and flourishing pre and post. As previously detailed, each program was evaluated through a pragmatic application of the RE-AIM framework (see Table 1 and Figure 3) (29, 31).

**Table 1:** Application of RE-AIM dimensions across three mindfulness programs.

	**Mindful Meet-ups**	**MUSCLE 2-day Retreat**	**MUSCLE 9-week Program**
Reach	~80 participants	73 participants	17 participants
Total Proportion	80/130 (61.5%)	73/130 (56.2%) (~33% attended at least 1 session)	17/130 (13%)
Representativeness	Family and Consumer Sciences (FCS) and Family Nutrition Program (FNP)	Limited participation from those outside FCS	Only FCS agents
**Deciding Factor: Mindful Meet-ups has the greatest proportional reach**			
Effectiveness	Unknown	Participants qualitatively reported that they felt valued within VCE after the retreat	No statistically significant change in flourishing or yoga self-efficacy
**Deciding Factor: Evaluate Mindful Meet-ups as a method to increase flourishing, MUSCLE programs not statistically effective**			
Adoption	Hokie Wellness partnered with original leading team to increase their reach	No outside adoption	No outside adoption
**Deciding Factor: Other teams and systems want to adopt Mindful Meet-ups**			
Implementation	Low resource investment, no curriculum to implement with fidelity	Medium time investment, no curriculum to implement with fidelity	High time investment, developed curriculum needs to be implemented with fidelity
**Deciding Factor: Mindful Meet-ups are easiest to implement with limited resources**			
Maintenance	Mindful Meet-ups have continued for multiple semesters	One time event	One time program
**Deciding Factor: Evaluate how other teams and systems adopting Mindful Meet-ups can improve maintenance**			

## Results

### Reach

*Phase I:* ~120 participants attended Mindful Meet-ups each week during the spring of 2020, declining to ~80 participants each week for the following semesters. The overall agent denominator of VCE is 220 for a proportion of 54.5% in the first semester and ongoing reach of 36.3% for the remaining two semesters. A majority (51%) of participants were from Family and Consumer Science and the Family Nutrition Program. Phase I was considered 4 for reach.

*Phase II:* The MUSCLE 2-day retreat had 73 individuals registered, and an average attendance of 26 on the day 1 sessions and 19 on the day 2 sessions. The proportional reach of MUSCLE 2-day was based on two denominators: (1) all agents in VCE and (2) agents who attended Mindful Meet-ups, for 33.2 and 91.3%, respectively. Phase II was considered a 3 for reach.

*Phase III*: Seventeen (13% of those eligible) participants completed the 9-week MUSCLE program. Phase III was considered a 1 for reach.

### Effectiveness

The 9-week MUSCLE program was not found to statistically increase flourishing, physical activity, or yoga self-efficacy (see Table 2). Mindful Meet-ups effectiveness is unknown due to low survey response rates (3 participants completed both pre- and post-surveys). All three iterations received a score of 3 due to positive qualitative feedback given on anonymous surveys for MUSCLE and positive feedback *via* emails and administration for Meet-ups (see Figure 3).

**Table 2:** Phase III (MUSCLE 9-week) program results.

	**Mean**	**Standard Deviation**	***p*-value**
**Flourishing (*****n** **=*** **11)**			
Pre Post	82.91 92.64	18.29 12.88	0.073
**Physical Activity (*****n** **=*** **11)**			
Pre Post	3.4 3.13	1.18 0.99	0.433
**Yoga Self-Efficacy (*****n** **=*** **11)**			
Pre Post	87 83.75	12.73 10.90	0.327

### Adoption

Currently no other systems or teams have adopted any of these low-dose iterations of Extension flourishing interventions. However, during Phase I, Hokie Wellness (the on-campus employee health promotion entity), delivered half of the sessions during semester 2 and 3.

### Implementation

The 2-day MUSCLE retreat (Phase II) required ~60 h of work over 6 weeks, including the 8 h each day of the retreat itself. The MUSCLE 9-week program (Phase III) required ~55 h of work for the primary team member over 15 weeks, or ~3 h and 40 min per week. Mindful Meet-ups (Phase I) require only 30 minutes to 1 h per week to deliver, based on if there is a handout for the week or only experiential learning. Both the 9-week MUSCLE program and the 2-day MUSCLE retreat were more labor-intensive programs, as they required more effort from the leading team to implement (see Figure 3 for implementation comparisons). Phase I was considered a 5, or “low implementation costs needed”. Phase II was rated a 3, and Phase III was considered a 1, or “high implementation costs”. The programs had no monetary cost associated with them.

### Maintenance

The MUSCLE 9-week program (Phase III) and the 2-day MUSCLE retreat (Phase II) have not been repeated. However, there have been requests from both past participants and those who did not attend the MUSCLE program or retreat to have it held again. The Mindful Meet-ups (Phase I) had support for over 1.5 years from the administration of VCE FCS/FNP as well as Hokie Wellness, however did not receive any support or feedback from administration for a fourth semester. The program leaders for Mindful Meet-ups did receive a number of unsolicited emails from participants speaking about how much they enjoyed the program, how useful it was for them, and how they had grown in mindfulness. One notable email stated, “This was a great program. The program was very informative in helping me become more centered with all the new normals in effect.”

Responses on the pre- and post-semester surveys for Mindful Meet-ups were highly limited, despite 58 people registering to attend the sessions and average attendance in the last semester being 13 (range 5–21). There were 9 pre-semester surveys completed and 6 post-semester surveys completed, with only 2 participants completing both the pre-and post- surveys. As Mindful Meet-ups had the longest maintenance of all three programs, it was scored as a 4.5 on maintenance, while both MUSCLE variations were scored as a 1, or “no maintenance” for maintenance. See Table 1 and Figure 3 for the application of RE-AIM across all three programs.

## Discussion

Throughout the COVID-19 pandemic, needs for mindfulness, physical activity, and human connection evolved rapidly. While many others have explored the need for yoga and mindfulness offerings during the COVID-19 pandemic (45–48) there is less available evidence for how mindfulness programs that were deemed efficacious prior to the pandemic were adjusted and implemented during the pandemic. Ongoing evaluation of programs is necessary to understand adaptations, sustainability, and impact (30, 49). Prior to the start of the pandemic, interventions deemed effective (including some discussed above) (3, 5, 19) often have very little follow-up information readily available. In contrast, through the application of a pragmatic approach, this study reports high reach and information on implementation and maintenance, but less empirical data on efficacy.

Extension specific Mindful Meet-ups sprung from a desire to connect while working from home in the earliest weeks of the pandemic, and continued to serve throughout the following semesters. The MUSCLE programs, originally conceived prior to the pandemic, pivoted and flowed online and ended up reaching a larger proportion of the target audience than initially thought would be possible. Utilizing the APDER process (29), the program leaders were able to assess what would best serve employee wellness and learning in that moment, and then quickly evaluate and change as needed. This is a valuable pragmatic approach to intervention implementation, as clinically studied interventions often take 15–17 years to be implemented (50, 51). While this study was not successful in fully capturing cross-sectional or longitudinal data on effectiveness, data that were available were positive and promising. This challenge is echoed the fact that “not everything that matters can be measured” (52). Investigative teams need to determine what dimension(s) of RE-AIM are most important for the next line of investigation and decision-making (29, 53). In the case of programs to promote flourishing among Extension agents, ongoing adoption and integration into worksite wellness offerings is the top priority. If the programs are not delivered, their other impacts can never be measured.

While the MUSCLE programs, in particular the 2-day retreat, were well attended, interest in conducting/attending them again has waned. This may be in part due to increased in-person activities, a proliferation of mindfulness and wellness based programming, or general disinterest in more mindfulness and physical activity content. When the last semester of Mindful Meet-ups came to a close, there was interest in continuing them from the participants, but due to shifting availability in the program leaders and wider availability of other, similar programs, Mindful Meet-ups was also discontinued. Participants from both the MUSCLE and Mindful Meet-ups who have expressed desire to attend more mindfulness and physical activity-based programming have been directed to other, on-going efforts that are not employee based and instead serve the general public. Some of these efforts include programs delivered by Hokie Wellness, who had partnered for the delivery of the Mindful Meet-ups program. Future research might examine the utilization of these public facing wellness programs as part of employee wellness opportunities and evaluate the effectiveness and need for employee-specific wellness programs.

## Conclusion

The RE-AIM and APDER cycles can be used before, during, and after implementation to evaluate programs and interventions for appropriateness, effectiveness, and necessity. These are especially helpful as guides when circumstances are changing rapidly, as in a pandemic. While employees expressed appreciation and personal commitment to attending mindfulness and physical activity-based programming throughout a variety of different programming iterations, there was no statistical improvement in flourishing or physical activity able to be discerned from the various programs described above. However, based on employee feedback on feeling valued and increasing their mindfulness practices, paired with the low cost and ease of implementation, employee wellness programs focusing on mindfulness are an important tool to consider when trying to increase employee satisfaction. Future studies may look at effectiveness of various styles of mindfulness programs and implementation and maintenance of those programs.

## Data Availability Statement

The datasets presented in this article are not readily available because of limited participant approval. Requests to access the datasets should be directed to AD, adysart@vt.edu.

## Ethics Statement

The studies involving human participants were reviewed and approved by Virginia Tech Institutional Review Board. The patients/participants provided their written informed consent to participate in this study.

## Author Contributions

AD and SH contributed to the conceptualization and design of the study. AD wrote the first draft of the manuscript with SH providing editing. Both authors contributed to manuscript revision, read, and approved the submitted version.

## Funding

This work was supported by the Virginia Tech Open Access Subvention Fund.

## Conflict of Interest

The authors declare that the research was conducted in the absence of any commercial or financial relationships that could be construed as a potential conflict of interest.

## Publisher's Note

All claims expressed in this article are solely those of the authors and do not necessarily represent those of their affiliated organizations, or those of the publisher, the editors and the reviewers. Any product that may be evaluated in this article, or claim that may be made by its manufacturer, is not guaranteed or endorsed by the publisher.
