# Supplementary material for: Mindfulness and Understanding of Self-Care for Leaders of Extension: Promoting Well-Being for Health Educators and Their Clients
Source: Front Public Health. 2022 May 13;10:862366. doi: 10.3389/fpubh.2022.862366 (PMC9136216; doi:10.3389/fpubh.2022.862366)
Supplement: Supplementary file 1 [file Data_Sheet_1.pdf]

Appendix A

Subject: Educational Sessions- Put a little MUSCLE into it

VCE is hosting educational sessions focusing on “Mindfulness and Understanding of Self-Care for Leaders of Extension” (MUSCLE). This is an experimental online program that is open the attendees of the Mindful Monday Meet-ups, PACE, and anyone else who would like to join. Participants will be asked to complete surveys before and after. These educational sessions will be focused on mindfulness, yoga, and self-care. For more information, contact adysart@vt.edu.

Appendix B

Flourishing Scale

VanderWeele, T. On the promotion of human flourishing. PNAS. 2017;114(31):8148-8156

Domain 1: Happiness and Life Satisfaction.
Overall, how satisfied are you with life as a whole these days?
0 = Not Satisfied at All, 10 = Completely Satisfied

In general, how happy or unhappy do you usually feel?
0 = Extremely Unhappy, 10 = Extremely Happy

Domain 2: Mental and Physical Health.
In general, how would you rate your physical health?
0 = Poor, 10 = Excellent

How would you rate your overall mental health?
0 = Poor, 10 = Excellent

Domain 3: Meaning and Purpose.
Overall, to what extent do you feel the things you do in your life are worthwhile?
0 = Not at All Worthwhile, 10 = Completely Worthwhile

I understand my purpose in life.
0 = Strongly Disagree, 10 = Strongly Agree

Domain 4: Character and Virtue.

I always act to promote good in all circumstances, even in difficult and challenging situations.
0 = Not True of Me, 10 = Completely True of Me

I am always able to give up some happiness now for greater happiness later.
0 = Not True of Me, 10 = Completely True of Me

Domain 5: Close Social Relationships.
I am content with my friendships and relationships.
0 = Strongly Disagree, 10 = Strongly Agree

My relationships are as satisfying as I would want them to be.
0 = Strongly Disagree, 10 = Strongly Agree

Domain 6: Financial and Material Stability.
How often do you worry about being able to meet normal monthly living expenses?
0 = Worry All of the Time, 10 = Do Not Ever Worry

How often do you worry about safety, food, or housing?
0 = Worry All of the Time, 10 = Do Not Ever Worry

Appendix C

<u>Yoga Self-Efficacy Scale Items</u>
Birdee et al. BMC Complementary and Alternative Medicine 2016;16(3)
DOI 10.1186/s12906-015-0981-0

Please circle the corresponding number for each item, with 1 being "strongly disagree" and 9 being "strongly agree"

Body
When I practice yoga…
1. I am able to remain as comfortable as possible while doing movements.
1 2 3 4 5 6 7 8 9
2. I am able to keep my mind focused on movements of my body.
1 2 3 4 5 6 7 8 9
3. I can coordinate the movements of my body with my breath.
1 2 3 4 5 6 7 8 9
4. I am able to move my body smoothly.
1 2 3 4 5 6 7 8 9
5. I am able to maintain a feeling of stability in my body.
1 2 3 4 5 6 7 8 9

Breath
When I practice yoga…
6. I am able to keep my breath smooth and continuous.
1 2 3 4 5 6 7 8 9
7. I am able to remain comfortable while regulating my breath.
1 2 3 4 5 6 7 8 9
8. I am able to focus my mind on my breath.
1 2 3 4 5 6 7 8 9
9. I am able to make my breath longer and deeper without feeling anxious.
1 2 3 4 5 6 7 8 9

Mind
During my yoga practice…
10. If distracted, I can re-focus my mind.
1 2 3 4 5 6 7 8 9
11. If asked, I am able to visualize or have an impression of an object in my mind.
1 2 3 4 5 6 7 8 9
12. I am able to remain focused on a meditative object or point.
1 2 3 4 5 6 7 8 9

Appendix D

Stanford Leisure-Time Activity Categorical Item

Kiernan M, Schoffman DE, Lee K, et al. The Stanford Leisure-Time Activity Categorical Item (L-Cat): a single categorical item sensitive to physical activity changes in overweight/obese women. Int J Obes (Lond). 2013;37(12):1597-1602. doi:10.1038/ijo.2013.36

During the past month, which statement best describes the kinds of physical activity you usually did? Do not include the time you spent working at a job. Please read all six statements before selecting one.

1. I did not do much physical activity. I mostly did things like watching television, reading, playing cards, or playing computer games. Only occasionally, no more than once or twice a month, did I do anything more active such as going for a walk or playing tennis.
2. Once or twice a week, I did light activities such as getting outdoors on the weekends for an easy walk or stroll. Or once or twice a week, I did chores around the house such as sweeping floors or vacuuming.
3. About three times a week, I did moderate activities such as brisk walking, swimming, or riding a bike for about 15–20 minutes each time. Or about once a week, I did moderately difficult chores such as raking or mowing the lawn for about 45–60 minutes. Or about once a week, I played sports such as softball, basketball, or soccer for about 45–60 minutes.
4. Almost daily, that is five or more times a week, I did moderate activities such as brisk walking, swimming, or riding a bike for 30 minutes or more each time. Or about once a week, I did moderately difficult chores or played sports for 2 hours or more.
5. About three times a week, I did vigorous activities such as running or riding hard on a bike for 30 minutes or more each time.

Almost daily, that is five or more times a week, I did vigorous activities such as running or riding hard on a bike for 30 minutes or more each time.
